# >ADVOCATING FOR ENGINEERING SOLUTIONS TO IMPROVE OLDER ADULT DRIVING LONGEVITY

**DOI:** 10.1093/geroni/igad104.2859

**Published:** 2023-12-21

**Authors:** Anne Dickerson

**Affiliations:** East Carolina University, Greenville, North Carolina, United States

## Abstract

To age-in-place, older adults need to access their community. As most older adults live in suburban/rural areas, driving is the primary and preferred means of mobility. While recent research has shown that drivers in the seventies have fewer crashes than middle-aged drivers, it is likely due to less exposure. However, it demonstrates that driving decisions need to be based on function, not age. Moreover, with the aging population and increase numbers of older adults with cognitive impairment and/or slowed processing speed, other strategies are needed to preserve and lengthen the driving lifetime. While efforts for developing alternative transportation options are working in some places, rural areas lack the infrastructure and potential for public transportation or volunteer programs beyond medical necessity. This presentation will present some roadway engineering proven countermeasures and community strategies that can protect and benefit aging drivers so they can drive longer while reducing crash risk. Stepping outside the typical gerontological community, this presenter became a contributing member of their state’s highway safety five-year plan. As an older driver advocate, I was successful in achieving older adults as an “emphasis” area in the present plan. For the next five-year plan, researching engineering solutions was deemed essential to advocate for specific strategies that protect both aging drivers and pedestrians. Since every state is required have a five-year plan for highway safety to receive federal funding, the presenter will outline successful strategies to participate in innovative ways for improving safety such as influencing engineering and department of motor vehicles partners.

